# Photo-Driven In Situ Solidification of Whole Cells through Inhibition of Trogocytosis for Immunotherapy

**DOI:** 10.34133/research.0318

**Published:** 2024-02-21

**Authors:** Hao Liu, Ke Huang, Hao Zhang, Xiaohui Liu, Hui Jiang, Xuemei Wang

**Affiliations:** State Key Laboratory of Digital Medical Engineering, School of Biological Science and Medical Engineering, Southeast University, Nanjing, Jiangsu 210096, China.

## Abstract

Achieving antitumor immunotherapy based on hybridization of multiple types of inactivated cells has attracted a lot of attention. However, the hybridized cells of disordered structure could result in the shedding of antigens and their transfer to immune cells, which suppresses tumor immunity through trogocytosis. Here, we report a strategy for in situ solidification of tumor whole cell by biomineralization for sustained stimulation of antitumor immunity. The near-infrared light was used to accelerate the breaking of Au=P bonds in auranofin, and the exposed Au atoms biomineralize at the secondary structure (β-corner) of the protein to form Au nanocrystals with in situ protein coronas in tumor cells. Au nanocrystals are anchored to the tumor cells through protein coronas, which fixes the morphology and antigens of whole tumor cells, rendering them physiologically inactive. Interestingly, this solidified tumor cell prevents immune cells from undergoing trogocytosis, which inhibits proximal and distal tumor growth. Thus, this study presents the strategy of solidified cells and its potential application in tumor immunotherapy.

## Introduction

Tumor therapeutic vaccines could induce specific immune responses in the body to treat or delay malignant tumors [1–3]. This treatment mode with low toxicity and sustained efficacy is superior to traditional chemotherapy and radiotherapy, which has attracted the attention of clinical community. The original studies were performed by extracting tumor cell membranes and re-encapsulating chemotherapeutic drugs as a therapeutic tumor vaccine [4–6]. As a consequence, the hybridization of tumor cell membranes or exosome membranes with other biological membranes has been used to stimulate organismal immunogenicity to enhance tumor immunotherapy, including bacterial membranes, blood cell membranes, and immune cell membranes [7–9]. Of note, whole-cell tumor vaccines show unique advantages due to their highly controllable preparation and low body immune rejection. Whole-cell tumor vaccines were obtained by electrocuting hepatocellular carcinoma cells in liquid nitrogen to eliminate pathogenicity while preserving their primary structure and chemotaxis to the lesion site [10]. Antigens are produced as a result of an external stimulus, without which there would be no immunotherapeutic effect. Tumor cells can be pretreated for better efficacy, such as the ability to genetically engineer the expression of specific antigens [11,12]. In situ tumor vaccines are based on a certain type of stimulated immune response to achieve a tumor immunotherapeutic effect and they can be obtained through in situ photothermal treatment of tumor tissues, photodynamic therapy, chemotherapy, radiotherapy, or sonodynamic therapy [13–16]. However, inactivated whole-cell tumor vaccines carry a high risk of causing recurrent cytokine storms or even unexpected Schwarzman reactions when administered multiple times to achieve antitumor effects.

Trogocytosis is a rapid process of cellular interaction that occurs in immunotherapy [17,18]. Immune cells, including T cells and natural killer (NK) cells, physically nibble off a portion of the tumor cell membrane at the stage of identifying the tumor cell, transferring antigens from the cell membrane of the target cell to the surface of the immune cell [19,20]. After the antigen is transferred to immune cells that do not express the antigen themselves, it can lead to mutual killing of immune cells, inhibit the effectiveness of immunotherapy, and promote tumor growth and metastasis [21,22]. It is important to note that trogocytosis is more likely to occur in broken tumor cells and outer vesicles after tumor treatment. This is one of the reasons for the poor prognosis of patients with malignant tumors [23]. This implies a potential antitumor negative regulatory mechanism for recombinant hybrid biofilms or inactivated tumor cells that do not have a stable cell membrane structure.

Biomineralization is the process by which inorganic matter ions undergo nucleation, crystallization, and growth in the presence of proteins to form inorganic matter [24,25]. Recently, patients with biomineralized calcium carbonate nanoparticles found within the tumor tissues had a significantly higher survival rate compared to patients without these nanoparticles [26,27]. Tang’s group exploits the fact that folic acid residues can induce calcium ion calcification on the surface of tumor cells, which can effectively inhibit tumor growth and metastasis without damaging normal cells [28–30]. Based on this seminal research, biomineralization of other ions has been applied in tumor therapy, such as iron (Fe), gold (Au), and copper (Cu) [31–33]. Biomineralization of platinum nanoparticles in the bloodstream enables long-lasting immune stimulation for the treatment of leukaemia [34]. The use of microbial in situ biomineralization of gold nanoparticles enables photothermal immunotherapy of tumors [35,36]. Alternatively, in situ biomineralization of iron oxide nanoparticles by Prussian blue can be used to obtain in situ tumor vaccines [37]. In our previous study, Au nanocrystals were found to biomineralize in tumor cells, and protein coronas were present on the surface of Au nanocrystals [38,39]. Therefore, it is reasonable to expect that biomineralization of Au nanoparticles may immobilize antigens, inhibit trogocytosis, and facilitate tumor immunotherapy.

**Fig. 1.**
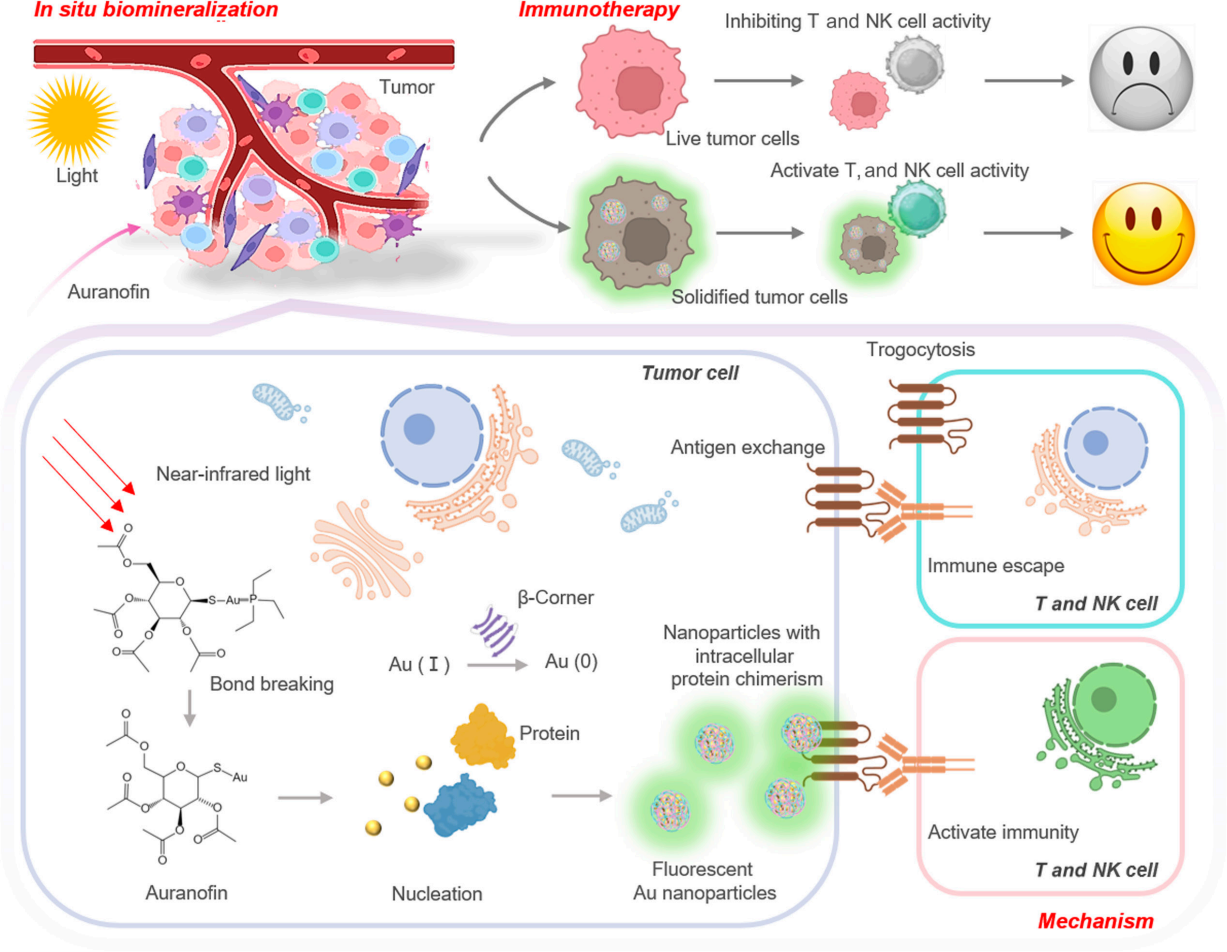
Schematic diagram of cell solidification process and immunotherapy mechanism based on biomineralization.

In this study, we have proposed a strategy based on the biomineralization of intracellular Au nanoparticles to anchor tumor cells for tumor immunotherapy (Fig. 1). Auranofin is a Food and Drug Administration-approved complex containing Au atoms which have been selected as a precursor for biomineralization. It could provide an opportunity to avoid the potential biosafety problems caused by commonly used chlorinated gold. The effect of near-infrared light (NIR) on the structure of auranofin and the morphology and properties of biomineralized Au nanoparticles within tumor cells were explored. Next, the morphological characteristics of the fixed tumor cells and the changes in whole proteins are illustrated. It reveals the mechanism by which fixed cells inhibit trogocytosis and enhance immunotherapy. Finally, the efficacy and safety of this strategy for distal tumors was explored. Thus, this study implies that biomineralization strategies have immense potential in the application of immunotherapy.

## Results and Discussion

### Biomineralization of intracellular Au nanoparticles

**Fig. 2.**
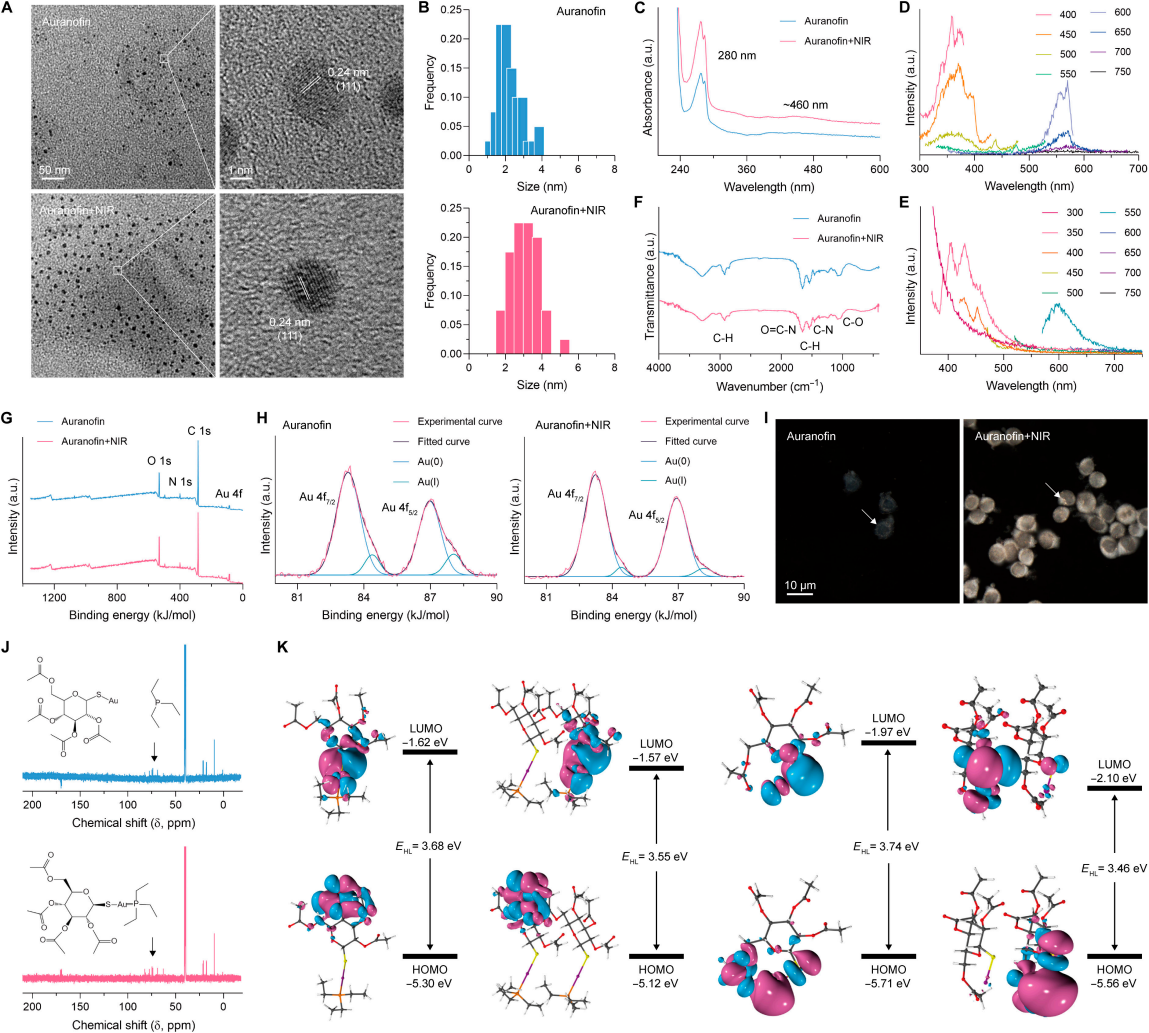
The characteristics of intracellular Au nanoparticles. (A) TEM and HRTEM image. (B) The size of Au nanoparticles after different treatments. (C) UV-vis absorption spectra. (D and E) Fluorescence excitation and emission spectra. (F) FT-IR spectra. (G and H) XPS and high-resolution XPS of Au 4f spectra. (I) Dark-field microscopic imaging of cells with different treatment. The concentration of auranofin is 100 μM. (J) NMR carbon spectra of auranofin before and after NIR irradiation. Blue line represents auranofin+NIR group. (k) DFT calculation of HOMO (bottom) and LUMO (top) of auranofin as a monomer or dimer before (left 2 columns) and after (right 2 columns) NIR irradiation.

HepG2 cells were employed to study the biomineralization of Au nanoparticles by auranofin. After treatment of auranofin, the cells were immersed in cell lysate and gold nanoparticles were obtained through high-speed centrifugation. The transmission electron microscopy (TEM) and high-resolution TEM (HRTEM) images were performed to obtain morphological and size characteristics of Au nanoparticles. As shown in Fig. 2A, Au nanoparticles exhibit uniform size distribution, and there seems to be a layer of colloid around the particles. Its lattice spacing is 0.24 nm, which belongs to the crystal characteristics of Au nanocrystals. There is no significant difference in the morphology and lattice spacing of Au nanoparticles with additional NIR, but the size is relatively large. The size of Au nanoparticles was quantified in TEM images (Fig. 2B). The average size of Au nanoparticles is 3.09 and 2.22 nm with or without treatment with NIR, respectively. The quantification results are consistent with the results of TEM visualization. The size of Au nanoparticles biomineralized by auranofin as a precursor is significantly smaller compared to gold chloride as a precursor, which may be related to the fact that the structure of auranofin inhibits the crystallization process [38]. Gold chloride without excessive chemical structure does not have spatial resistance, which could lead to further crystalline growth to larger sizes.

Next, ultraviolet-visible (UV-vis) absorption spectrum and fluorescence spectrum (FL) were chosen to analyze the optical properties of Au nanoparticles. The UV-vis absorption spectra demonstrated the presence of absorption peaks at 280 and 460 nm, and other absorption peaks were insignificant (Fig. 2C). The absorption peak at 280 nm is attributed to tryptophan in proteins, indicating that the Au nanoparticles have proteins embedded on their surface and could anchor proteins. Fluorescence excitation and emission spectrum was used to test the fluorescence effect of Au nanoparticles (Fig. 2D and E). The results showed that regardless of NIR treatment or not, Au nanoparticles exhibited 2 distinct excitation and emission peaks (Fig. S1A, Supplementary Materials). The fluorescence intensity is higher for Au nanoparticles treated by NIR, which indicates that NIR could promote the biomineralization of fluorescent Au nanoparticles. Since the wavelength of fluorescence excitation is same as the UV absorption peak, the light absorption may be released through the fluorescence pathway.

Fourier transform infrared spectrum (FT-IR), x-ray photoelectron spectrum (XPS) and its high-resolution XPS were performed to provide information on the chemical bonding of Au nanoparticles and the valence state of Au. The FT-IR spectrum results show vibrational peaks at 1,060, 1,250, 1,480, 1,700, and 3,100 cm^−1^ for Au nanoparticles that are attributed to C–O, C–N, C–H, O=C–H and O–H bonds (Fig. 2F). Since Au nanoparticles are biomineralized in a complex protein environment within tumor cells, a possible reason for the appearance of these vibrational peaks is the protein coronas and residues of auranofin formed on the surface of Au nanoparticles. The protein coronas formed may be one of the factors promoting tumor immunotherapy. The XPS results show that Au nanoparticles contain C, O, N, H, and Au elements, which corresponds to Au nanoparticles coated with protein coronas (Fig. 2G and Fig. S1B and C). The elemental types and chemical bonds are not affected by NIR. High-resolution XPS was taken to analyze the effect of NIR on the valence state of the Au element in Au nanoparticles. In Fig. 2H, the relative amount of Au(I) (about 4.5%) is significantly lower than Au(0) (about 95.5%) in Au nanoparticles treated with NIR, which is not the case for Au nanoparticles without NIR. This indicates that NIR could promote the transformation of auranofin from Au(I) to Au(0) in HepG2 cells and accelerate the biomineralization of Au nanoparticles. Dark-field microscopic imaging was applied to visualize the formation of intracellular Au nanoparticles in relation to NIR (Fig. 2I). It can be found that the number of Au nanoparticles in HepG2 cells treated with NIR is significantly enhanced than that without NIR treatment, which is consistent with previous conjectures. A positive correlation was observed between the concentration of auranofin and the number of intracellular Au nanoparticles (Fig. S2). Of interest, HepG2 cells were fixed while biomineralization was occurring, which was not observed in the low-concentration auranofin and without NIR groups.

In order to investigate the chemical mechanism of the influence of NIR on auranofin, the nuclear magnetic resonance (NMR) was used to analyze the structure of auranofin. Density functional theory (DFT) was used to calculate the lowest unoccupied molecular orbital (LUMO), highest occupied molecular orbit (HOMO) and HOMO-LUMO gap (*E*_HL_) of auranofin with different structures and aggregation degrees. The results of ^13^C-NMR show that NIR is able to stimulate the breaking of Au–P bonds, and the structure is consistent with the software prediction (Fig. 2J and Fig. S3). The LUMO and HOMO of the unchanged chemical structure of auranofin monomers are −1.62 and −5.30 eV, while those of the dimers are −1.57 and −5.12 eV, respectively (Fig. 2K). The *E*_HL_ of dimers is reduced from 3.68 to 3.55 eV compared to monomers. The LUMO and HOMO of the changed chemical structure of auranofin monomers are −1.97 and −5.71 eV, while those of the dimers are −2.10 and −5.56 eV, respectively. The *E*_HL_ of dimers is reduced from 3.74 to 3.46 eV compared to monomers. The *E*_HL_ of dimers is lower than that of monomers, which indicates that higher concentrations of auranofin are more prone to biomineralization. Importantly, it has a lower *E*_HL_ compared to that after NIR induced structural changes in auranofin. Importantly, auranofin containing Au–P bonds has a lower *E*_HL_ compared to those without Au–P bonds. This further explains the mechanism that high concentrations and NIR may promote the biomineralization of intracellular Au nanoparticles.

### Tumor cells solidify and preserve immunogenicity

**Fig. 3.**
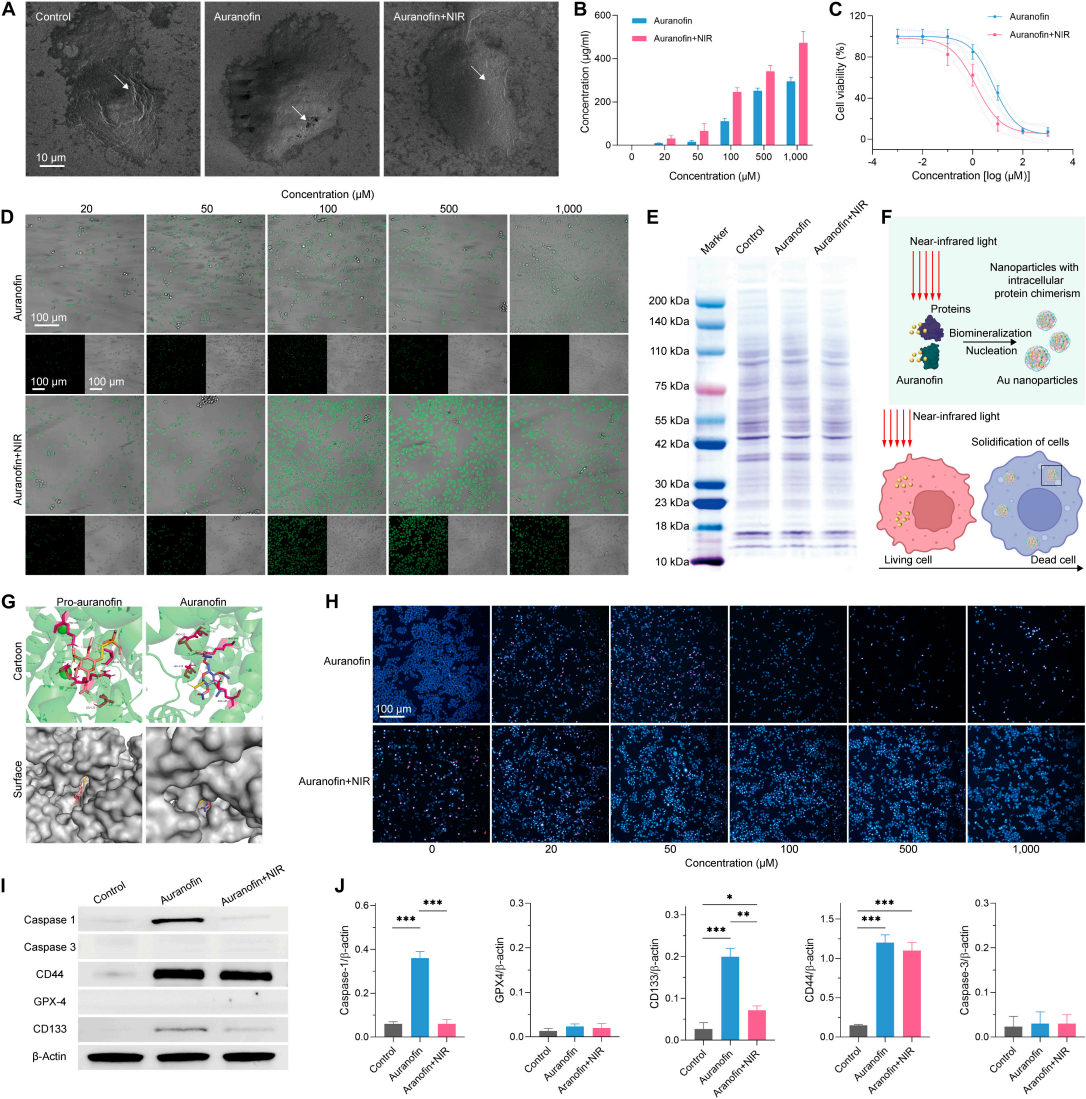
The characteristics of HepG2 cells after auranofin treatments. (A) SEM image. The insets belong to the corresponding selected area electron diffraction image. (B) ICP-MS of intracellular Au contents. (C) Cell viability after incubation with auranofin. (D) CLSM images of cells after incubation with auranofin. (E) Coomassie brilliant blue staining of whole-cell proteins. (F) Schematic diagram of the biomineralization and cell solidification mechanism of intracellular Au nanoparticles. (G) Molecular docking of auranofin and albumin. Bovine serum albumin is used as the standard protein. (H) Hoechst 33258 and PI staining of cells. (I and J) Western blot and the protein quantification. *P < 0.05, **P < 0.01, and ***P < 0.001 was analyzed by Student *t* test.

The cell solidification triggered by Au nanoparticle biomineralization was further validated. Scanning electron microscopy (SEM) was performed to obtain morphological characteristics of HepG2 cells after treatment with auranofin and NIR (Fig. 3A). The results showed that the cell membrane surface was smooth and structurally intact in the auranofin+NIR group, but its size seemed to be larger than the control group. Only the cell membranes treated by auranofin were structurally deficient, with occurrence of pores, while the overall structure was also the same as that of the control group. This indicates that the treatment of auranofin+NIR results in better immobilized cells without cell fragmentation. Inductively coupled plasma mass spectrometry (ICP-MS) was used to further validate that auranofin is uptaken into cells and causes biomineralization (Fig. 3B). The results are consistent with the theoretical conjecture of a positive correlation between the content of intracellular Au elements and the concentration of auranofin. Cell counting kit-8 (CCK-8) was used to observe the cell viability of the HepG2 cells with different treatment (Fig. 3C). The NIR contributed to the loss of cell viability, with its half maximal inhibitory concentration (IC_50_) from 6.727 to 1.356 μM. It is hinted that biomineralization of Au nanoparticles is responsible for the inactivation of tumor cells. Confocal laser scanning microscopy (CLSM) was applied to observe the cellular fluorescence produced by fluorescent Au nanoparticles (Fig. 3D and Fig. S4). The intracellular fluorescence intensity tends to increase with the increasing concentration of auranofin in the cells, which can be enhanced by treatment with NIR. Its cellular morphology is consistent with the results of SEM. It indicates that Au species are ingested by the cells to form fluorescent Au nanoparticles through biomineralization.

Whole-cell proteins were extracted, and polyacrylamide electrophoresis and Coomassie brilliant blue staining were used to analyze the impact of biomineralization on total proteins (Fig. 3E). The results show that the contents of proteins with different molecular weights in the treated cells are relatively unaffected. The possible reason is that the occurrence of biomineralization of such Au nanoparticles may be related to multiple proteins, which could lead to cell death through this method (Fig. 3F). Molecular docking was adopted to analyze the nucleation sites in proteins for the Au atoms in auranofin (Fig. 3G). Due to the fact that this unnatural biomineralization may be related to multiple proteins, albumin (take bovine serum albumin as a model) is used as a standard protein for molecular docking. The nucleation sites of auranofin are located in the molecular nests of proteins. The amino acids related to the nucleation site of auranofin treated with NIR are arginine (Arg), leucine (Leu), and proline (Pro). The amino acids related to auranofin are Arg, Leu, and histidine (His). It was found that the binding energy of auranofin+NIR to nucleation sites was 6.12, which was higher than that of auranofin (5.13). This suggests that gold atoms form Au nanoparticles by nucleating in protein-specific structures. The biomineralized Au nanoparticles can immobilize intracellular proteins. Notably, it does not continue to crystallize and growth by reason of spatial site resistance (Fig. 3F).

The integrity of cell membranes, types of death, and changes in surface antigens were further analyzed to determine the potential immunotherapeutic effects of this strategy. Hoechst 33258 staining of the nucleus is not affected by cell membrane permeability, while propidium iodide (PI) staining occurs only with permeable cell membranes. Hoechst 33258 and PI staining were used to exhibit that the cell membrane receives the effects of auranofin and NIR (Fig. 3H). The results show that high concentrations of auranofin cause rupture of the cell membrane, which is instead intact after additional NIR. The only phenotype of pyroptosis is cell membrane rupture, which suggests that auranofin may contribute to cell death through pyroptosis. The possible reason for the absence of cell rupture in the group with additional NIR irradiation is the biomineralization of Au nanoparticles. The intracellular proteins were immobilized with no signal pathways initiating cell death. It is worth noting that a complete cell membrane does not lead to the leakage of a large amount of cell contents, which may not result in an immune cytokine storm. This may be a prerequisite for further improving the safety of tumor immunotherapy. The Western blot was used to confirm our hypothesis (Fig. 3I and J). Protein markers of apoptosis, pyroptosis and ferroptosis are Caspase-3, Caspase-1, and GPX-4. Auranofin leads to Caspase-1 expression in HepG2 cells, but the opposite is true for that with NIR irradiation, thus corresponding to our hypothesis. Tumor cell markers CD44 and CD133 were selected to indicate the presence or absence of immobilized antigens. These antigens are present in the immobilized cells, presumably capable of good immunogenicity. In addition, the cell membranes were not ruptured in the NIR group, which may not have caused a risk of an immune factor storm.

### In situ tumor therapy through inhibition of trogocytosis

**Fig. 4.**
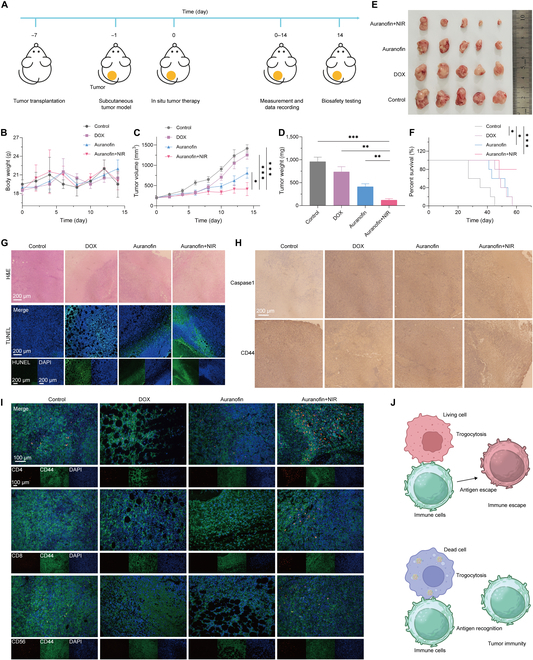
Therapeutic mechanism of unilateral back tumors in mice. (A) Schematic diagram of the therapy process. (B) Mice weight and (C) tumor volume in mice after treatment. (D) The weight and (E) digital photos of tumors. (F) Survival curve of mice. (G) H&E staining and TUNEL staining of tumor tissues. (H) Immunohistochemistry of caspase 1 and CD 44 after different treatments. (I) Immunofluorescence double staining of CD4, CD8, and CD56 (red fluorescence) and CD44 (green fluorescence), while DAPI exhibits blue fluorescence. (J) The mechanism of immune recognition induced by trogocytosis. *P < 0.05, **P < 0.01, and ***P < 0.001 analyzed by Student *t* test.

A Balb/c mice subcutaneous unilateral tumor model was constructed to assess the antitumor effects of the immobilized cell strategy based on Au nanoparticle biomineralization. After the tumor model was constructed successfully, intratumor injection of auranofin and in situ NIR irradiation were performed. During the treatment period, the tumor volumes and body weights were monitored and the mice tumors were removed and weighed on the last day (Fig. 4A). The body weight of the mice was not affected by the treatment process (Fig. 4B), suggesting that the tumor treatment method of auranofin+NIR does not cause extensive damage to the mice organism. As shown in Fig. 4C, auranofin and adriamycin (DOX) inhibit the growth of tumor within a short period of time after single intratumoral injection. The tumor volume was maintained at around 350 mm^3^ after auranofin+NIR treatment. The results indicate that auranofin+NIR fixes the cells through biomineralization and does not lead to sustained cell growth. On the last day of the observation cycle, mice tumors were removed for weighed and photographed. Normal tissues were fixed in paraformaldehyde and whole blood was removed and stored at −80°C for subsequent biosafety analysis. The results of tumors weight and photograph (Fig. 4D and E) were consistent with the tumor volume results. In addition, parallel groups of tumor model mice were used to analyze the effect of auranofin on survival in mice (Fig. 4F). The feasibility of this biomineralization strategy was demonstrated by the fact that auranofin+NIR prolonged survival in mice compared to auranofin and DOX. The survival rate of mice treated with auranofin was not as high as that of mice treated with auranofin+NIR. The possible reason is that NIR promotes the biomineralization of auranofin, leading to the fixation of gold atoms at the tumor site. This can effectively avoid side effects such as heavy metal poisoning caused by auranofin, and achieve safer tumor immunotherapy.

Pathological sections and stains are exploited to study the efficacy of tumor therapy and the mechanisms of immunotherapy. The hematoxylin and eosin (H&E) staining results showed that the tumor cells in the control group show diffuse growth, with overlapping nuclei and dense and disordered arrangement. The cytoplasm of cancer cells was abundant, with obvious atypia, and typical pathological nuclear division phenomenon was visible (Fig. 4G). Tumor cells had a reduced number of cells, a reduced nucleoplasmic ratio, reduced nuclear division phenomena, pale cytoplasm, pale nuclei, and small nucleolus size in the auranofin+NIR group. However, its cell membrane structure is intact. The auranofin+NIR immobilizes and inactivates tumor cells through biomineralization. Terminal-deoxynucleoitidyl transferase mediated nick end labeling (TUNEL) staining exhibited the same results. In Fig. 4H, immunohistochemistry was used to verify that the mode of tumor cell death in vitro was consistent with in vivo. The results revealed that tumor tissues with auranofin+NIR overexpressed Caspase-1, which is consistent with the results of in vitro. Moreover, it can better preserve the tumor antigen, CD44 protein. Thus, it is suggested that auranofin+NIR promotes tumor cell immobilization by means of pyroptosis but preserves cell membrane integrity and antigenicity. It can promote immune cell recognition and achieve tumor immunotherapy.

Immunofluorescence double staining was employed to determine the extent of immune cell aggregation and the occurrence of trogocytosis in tumor tissues (Fig. 4I). CD44 serves as a tumor antigen, CD4 and CD8 serve as T lymphocyte markers, and CD56 serves as a marker for NK cells. When cellular trogocytosis occurs, tumor cell antigens are transferred to the immune cell membrane, which leads to the appearance of green fluorescence used to label tumor antigen CD44 on immune cells, which overlaps with the red fluorescence labeling of immune cells, resulting in yellow fluorescence. The occurrence of trogocytosis was determined by observing this phenomenon. The results showed that the immobilized cells (by auranofin+NIR) were able to enrich immune cells in tumor tissues, including CD4^+^ T cells, CD8^+^ T cells, and NK cells. The immune cells cannot be accumulated in tumor tissues treated with chemotherapy drugs, which may inhibit immune function compared to the control group. When trogocytosis occurs, immune cells transfer part of the tumor cell membrane to the immune cell membrane (Fig. 4J). The red fluorescent labeled immune cell markers (CD4, CD8, and CD56) overlap with the green fluorescent labeled tumor cell markers (CD44), which results in the appearance of yellow fluorescence. As shown in Fig. 3I, trogocytosis does not occur in CD4^+^ T cells and CD8^+^ T cells in the auranofin+NIR group. Compared to the control group, although auranofin+NIR inhibited NK cells from trogocytosis, it was not a complete inhibition. Therefore, the strategy of cell immobilization could significantly inhibit immune cells from trogocytosis, thus approaching tumor immunotherapeutic effects and achieving prolonged immune stimulation (Fig. 4J).

### Immunotherapy

**Fig. 5.**
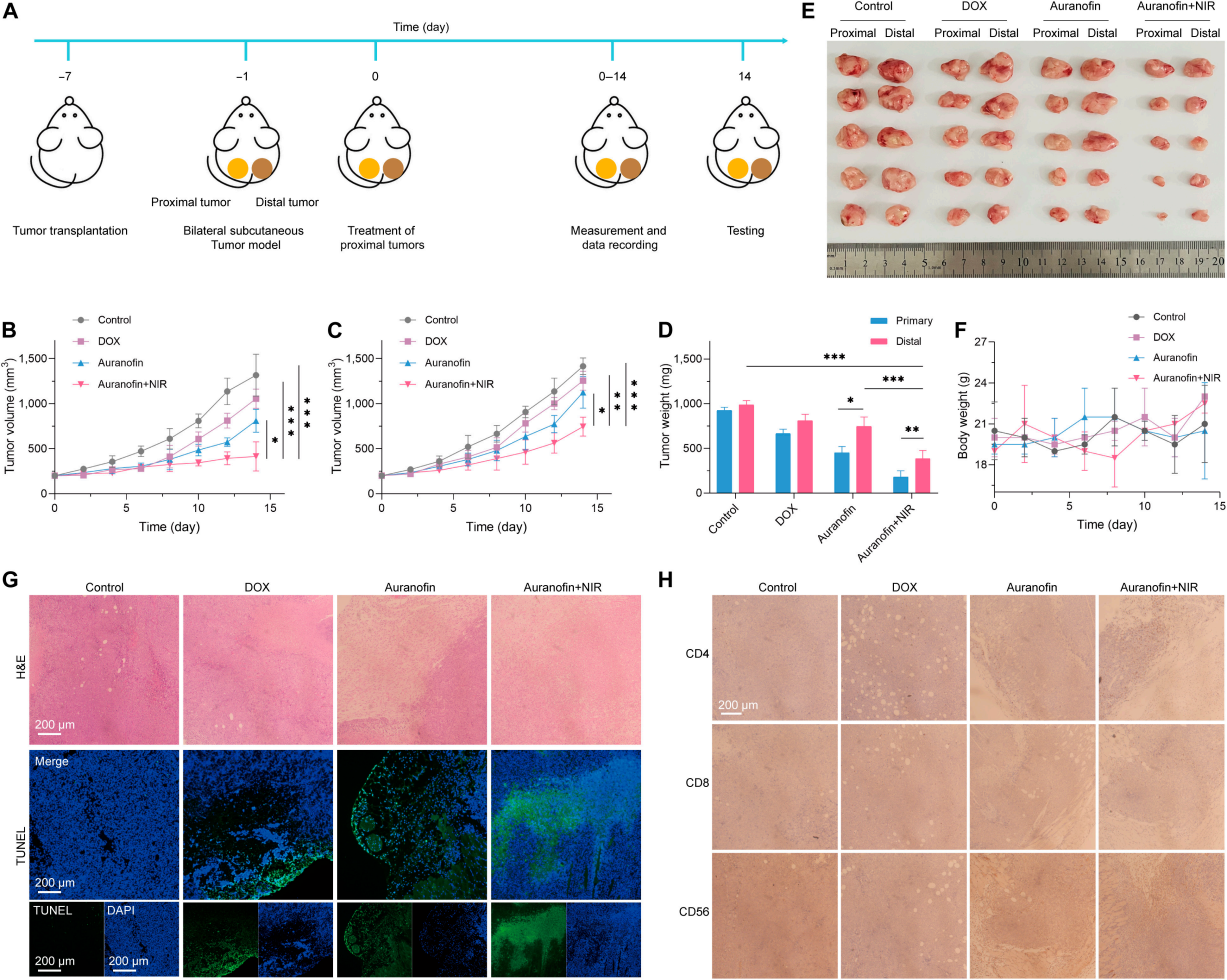
Immunotherapy for mice tumors. (A) Schematic diagram of the immunotherapy process. (B and C) The volume of proximal and distal tumors. (D) The quality of mice tumors. (E) Digital photos of mice tumors. (F) Mice weight. (G) H&E staining and TUNEL staining of tumor tissues. (H) Immunohistochemistry of CD4, CD8 and CD56 after different treatments. *P < 0.05, **P < 0.01, and ***P < 0.001 analyzed by Student *t* test.

The immunotherapeutic effect of the cell immobilization strategy on distal tumors was further studied by building a subcutaneous double tumor model in Balb/c mice (Fig. 5A). The methodology was consistent with the process of unilateral tumor treatment, monitoring the volume of bilateral tumors, mice body weight, etc., during treatment. The results show that the therapeutic effect on proximal tumors is consistent with that of unilateral tumors, with the ability of auranofin+NIR to inhibit the increase in tumor volume (Fig. 5B). After treatment, the volume of the distal tumor decreases to various degrees, most significantly in the auranofin+NIR group (Fig. 5C). This indicates that immobilized cells can stimulate the immune system in mice and have a certain immunotherapeutic effect on distant tumors. The reason may be attributed to the fact that the trogocytosis of immune cells is inhibited in the auranofin+NIR group. On the 14th day, the tumor was removed, weighed, and photographed (Fig. 5D and E). The volume and weight of distal tumors were suppressed in the DOX and auranofin groups but not as markedly as in the auranofin+NIR group. During the treatment process, the weight of the mice was not affected (Fig. 5F). This indicates that this strategy has a certain degree of biosafety.

Pathological testing was used to explain the mechanism by which distal tumors are suppressed. The H&E staining results show that in the distal tumor tissues of the auranofin+NIR group, cells disperse and shrink, presenting a state of cell death. This phenomenon is not significant in the distal tumor tissues of the DOX and auranofin groups (Fig. 5G). The TUNEL fluorescence staining show that nucleic acid in the nucleus is broken in the distal tumor tissues of auranofin+NIR group, which indicates that the distal tumors of auranofin+NIR group are effectively suppressed. Immunohistochemical staining was applied to determine the presence of an infiltration of immune cells in the distal tumor (Fig. 5G). The results reveal the presence of CD4^+^ T cells, CD8^+^ T cells and NK cells enriched in the distal tumor tissues of the auranofin+NIR group. Interestingly, no immune cell infiltration is found in the distal tumors of the DOX group, possibly due to the reason that chemotherapy suppresses the immune effects. Therefore, auranofin+NIR is able to stimulate immune cells through immobilization of cells for a long period of time and is able to achieve inhibition of distal tumors.

### Safety assessment

**Fig. 6.**
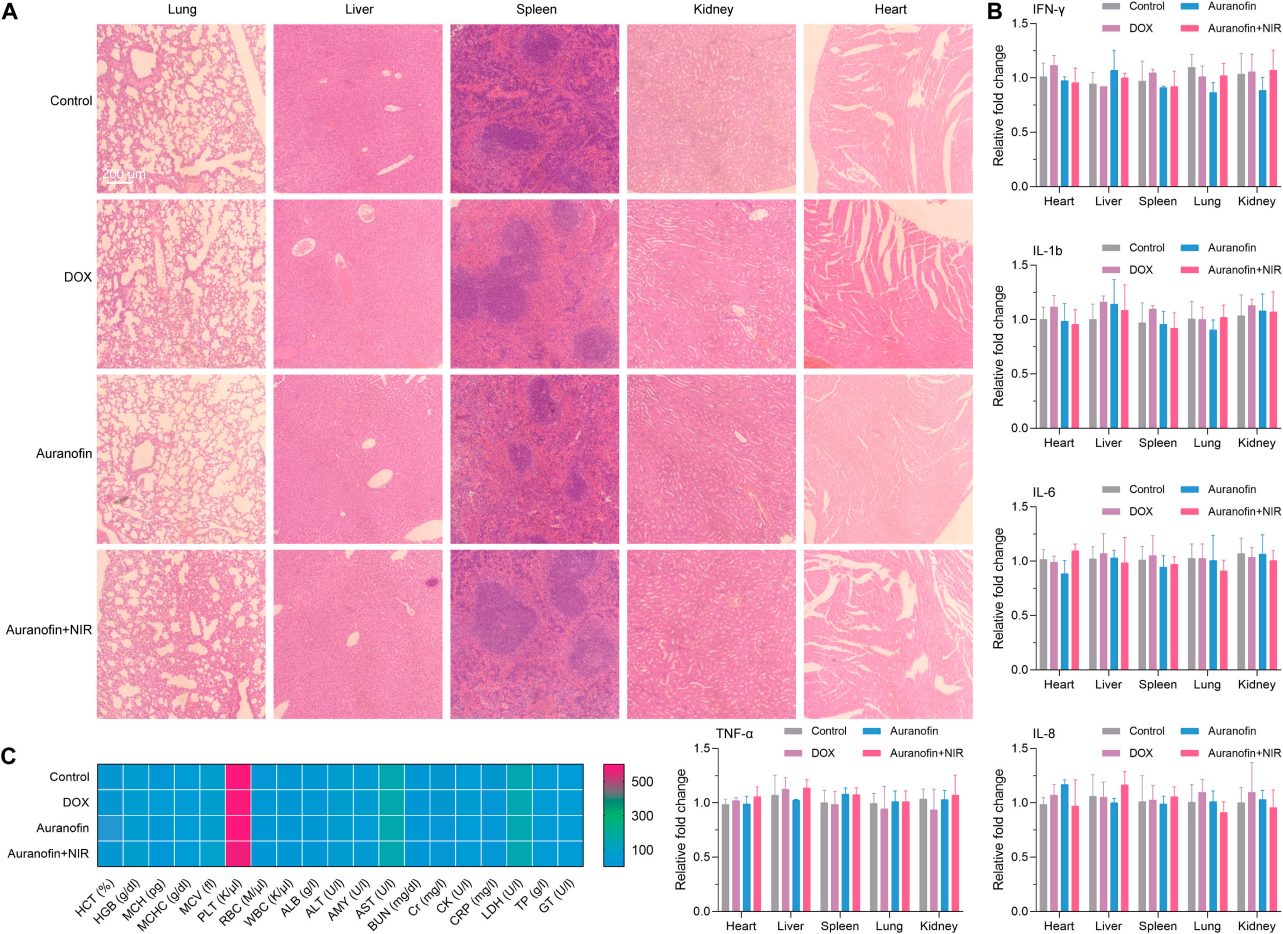
Safety assessment. (A) H&E staining. (B) The content of inflammatory factors in normal tissues. (C) Blood indicators.

On day 14 after treatment, major organs (kidney, liver, lung, heart, and spleen) and whole blood were collected from mice for safety evaluation. The H&E staining shows no significant cytoarchitectural changes or inflammatory lesions in the major organs (Fig. 6A). The expression changes of immunological factors (interferon-γ, interleukin-1b (IL-1b), IL-6, IL-8, and tumor necrosis factor-α) in treated samples were also not evident compared with normal tissues (Fig. 6B). This cell immobilization strategy does not cause a systemic storm of immune factors in mice. The mice blood analyses show that the blood indices are consistent with those of the control group after treatment, without changes in the composition and content of the mice’s blood (Fig. 6C). These results suggest that the strategy possesses feasibility for biological applications.

## Conclusion

In summary, we propose a strategy based on biomineralization to immobilize cells. The NIR could induce changes in the structure of auranofin, exposing Au atoms. Then they nucleate on intracellular proteins, forming fluorescent Au nanoparticles. The structure of genistein and proteins can lead to steric hindrance, which inhibits the crystallization and growth of fluorescent Au nanoparticles. Biomineralized Au nanoparticles contain chimeric proteins, which can immobilize tumor cells and lead to their death, but maintain the integrity of antigens and cell membranes in tumor cells. Importantly, the immobilized cells inhibit immune cells from undergoing trogocytosis, which allows immune cells to be enriched at the tumor site. It has an immunotherapeutic effect on distal tumors that does not cause immune factor storm and other side effects. Thus, this finding reveals the phenomenon and mechanism by which biomineralization can induce cellular immobilization and confirms the promise of biomineralization strategies for biomedical applications.

## Materials and Methods

### Materials

Auranofin (CAS 34031-32-8), CCK-8, Dulbecco’s modified Eagle medium, fetal bovine serum, phosphate buffer (PBS), ethanol, dimethylbenzene, Hoechst 33258, 4',6-diamidino-2-phenylindole (DAPI), PI, polyformaldehyde, nitric acid, and paraffin were purchased from Aladdin (Shanghai, China). Coomassie brilliant blue (G-250, CAS 6104-58-1), agarose gel electrophoresis kit (Beyotime Biotechnology), and sodium dodecyl sulfate polyacrylamide gel electrophoresis gel preparation kit (Beyotime Biotechnology) were purchased from Beyotime (Shanghai, China). All antibodies were purchased from Abcam. Ultrapure water (18.2 MΩ, Millipore Co, USA) was used in all of the experiments.

### Characterization of Au nanoparticles

The morphologies of the HepG2 cells were observed by SEM (Hitachi, Japan). The morphologies of the Au nanoparticles were measured by TEM (JEM-2100F, JEOL). The FL, FT-IR, XPS and UV-vis absorption spectra of Au nanoparticles were obtained by a fluorescence spectrometer (Duetta, HORIBA), Fourier transform infrared spectrometer (Nicolet iS5, Thermo Fisher Scientific), x-ray photoelectron spectrometer (ESCALAB 250, Thermo Fisher Scientific), and UV-vis spectrophotometer (UV6100, MAPADA). The dark-field image and fluorescence image of HepG2 cell were measured by CLSM (A1R HD25, Nikon). The images of pathological sections were captured using a fluorescence microscope (Axio Imager 2, ZEISS). The element content of HepG2 cell was investigated with inductively coupled plasma mass spectrometer (Ultima Expert LT, HORIBA). The microplate reader (Multiskan, Thermo Fisher Scientific) was used to measure cell viability. Gel imaging system is used for Western blot image (E-Gel Imager, Thermo Fisher Scientific). The NMR spectra of auranofin before and after treatment were tested by an NMR spectrometer (Avance 500 MHz, Bruker).

### Biomineralization of intracellular au nanoparticles

HepG2 liver cancer cells come from the Shanghai Cell Bank (China). Fetal bovine serum (10%) and high-glucose Dulbecco’s modified Eagle medium were used to culture HepG2 cells under 37 °C and 5% CO_2_. When the cell coverage in the culture plate reaches 70%, the complete culture medium was removed and the cells were washed twice with PBS. Then, PBS containing different concentrations (0, 20, 50, 100, 500, and 1,000 μM) of genistein were used to incubate HepG2 cells. The wavelength range of NIR used to treat auranofin, cells, and tumor tissues is 500 to 800 nm, with a power of 1 W/cm^2^ and an irradiation time of 20 min. The cells were continuously cultivated for 3 d to obtain biomineralized cells. Cell lysate was applied to lyse the cells, and cell debris was removed by centrifugation at 3,000 g for 10 min. The fluorescent Au nanoparticles were collected from the cells by centrifugation at 12,000 g for 10 min. It is stored in a −80 °C refrigerator for subsequent TEM, UV-vis absorption spectra, FL spectra, FT-IR spectra, XPS, and other tests.

HepG2 cells in normal culture were used as the control group. SEM, CLSM, and dark-field microscopy are used to obtain fluorescence imaging and dark-field imaging of HepG2 cells. Hoechst 33258 and PI were taken to stain the HepG2 cells nuclei treated with different concentrations of auranofin and NIR to analyze whether the cell membrane was ruptured. Before and after being irradiated with NIR light for 20 min, the chemical structure of auranofin was determined by NMR. ICP-MS was applied to make sure that the enrichment of Au nanoparticles inside the cells.

After different treatment, the buffer was removed. PBS was used to flush the cells. CCK-8 was used to incubate cells for 1 h, and it was measured by a microplate reader at 450 nm.

### Protein analysis of cells

The cells treated with auranofin and NIR were collected by cell scrapers into centrifuge tubes. Cells were collected by centrifugation at 2,000 g for 10 min, and a small amount of cell lysate was added to the precipitate to obtain intracellular proteins. Polyacrylamide gel electrophoresis was used to separate proteins of different molecular weights, and Coomassie Brilliant Blue was used to stain the protein bands for photographs. A digital camera was used to acquire strip images to determine the integrity of cured intracellular proteins.

Intracellular protein quantification results were obtained by Western blot assay. The specific experimental steps can be referred to previous literatures [40–42]. The primary antibodies were CD44, CD133, GPX-4, Caspase-1, Caspase-3, and β-actin. The dilution ratio of primary antibody is 1/500, and the dilution ratio of secondary antibody is 1/5,000. The raw gray values are automatically read by the software and the relative content is assessed by the ratio of the indicator gray value to the internal reference gray value.

### Molecular dynamic simulation

DFT was exploited to acquire LUMO, HOMO, and HOMO-LUMO gap (*E*_HL_) for auranofin. Geometry optimization and frequency analysis are performed in the B3LYP hybrid function. Molecular orbitals are analyzed by Multiwfn 3.83 and VMD v 1.9.3 molecular visualization software.

To calculate the interaction between auranofin and protein molecules (bovine serum albumin), dynamic simulations and molecular docking were performed. Auranofin was geometrically optimized in the Autodock software. Geometric configurations of proteins were downloaded from public databases (Protein Data Bank) and geometric optimization was implemented. Next, molecular docking is performed to obtain the binding energy and docking structure, with visualization by PyMOLWin software.

### Animal experiments

The male Balb/c mice were obtained from Zhongda Hospital Laboratory Animal Centre, Southeast University. Animal experiments were approved by the Animal Ethics Committee of Zhongda Hospital, Southeast University, and were conducted according to the guidelines for animal welfare and protocols (82061148012). H22 cells were purchased from the Shanghai cell bank and cultured in a culture bottle. Serum free culture medium was used to resuspend H22 cells, which were then implanted into Balb/c mice to construct a subcutaneous tumor model. The dual-tumor subcutaneous model involves injecting H22 cell suspension on the other side of the mice’s back. The Balb/c mice were kept for about 1 week, and the subcutaneous tumor volume reached 200 mm^2^, suggesting the successful construction of Balb/c mice subcutaneous tumor model.

There were 4 separate groups, including control, doxorubicin (DOX), auranofin, and auranofin+NIR. The treatment method for the control group was to inject 50 μl of PBS into the tumor site. The DOX group received in situ injection of 3 mg/g of DOX, while the auranofin group received in situ injection of 5 mg/g of auranofin. The auranofin+NIR group received in situ injection of 3 mg/g of auranofin with NIR irradiation. After 48 h of drug treatment, the mouse tumor was taken and fixed in paraformaldehyde for subsequent pathological analysis. The mice weight and tumor volume were measured every other day. After the 14th day, the Balb/c mice tumor was weighed and photographed. Normal tissues were sectioned for H&E staining and immune factor analysis, and whole blood of mice was taken out for analysis.

### Pathology analysis

The fixed tissue was sliced, dewaxed, and permeable. Then, H&E were used to stain the sections. The TUNEL staining kit is used to investigate nucleic acid cleavage in tumor cells. The detailed steps of immunohistochemistry and immunofluorescence double labeling refer to previous literature [40]. The primary antibodies were CD44, Caspase-1, CD4, CD8, and CD56. The dilution ratio of primary antibody is 1/1,000 and the dilution ratio of secondary antibody is 1/2,000. After staining, the slices were preserved with sealing agent and observed using a fluorescence microscope.

## Data Availability

All data that support the findings of this study are available from the corresponding author upon reasonable request.
