# Supplementary material for: Photo-Driven In Situ Solidification of Whole Cells through Inhibition of Trogocytosis for Immunotherapy
Source: Research (Wash D C). 2024 Feb 21;7:0318. doi: 10.34133/research.0318 (PMC10879965; doi:10.34133/research.0318)
Supplement: Supplementary 1 — Figs. S1 to S4 [file research.0318.f1.pdf]

Supporting Information

Photo-driven in situ solidification of whole cells through inhibition of trogocytosis for immunotherapy

Hao Liu,[1] Ke Huang,[1] Hao Zhang,[1] Xiaohui Liu,[1,*] Hui Jiang,[1,*] and Xuemei Wang,[1,*]

[1] State Key Laboratory of Digital Medical Engineering, School of Biological Science and Medical Engineering, Southeast University, Nanjing, Jiangsu 210096, China

[*] Corresponding author:

xuewang@seu.edu.cn (Dr. Xuemei Wang); sungi@seu.edu.cn (Dr. Hui Jiang); 101013182@seu.edu.cn (Dr. Xiaohui Liu)

Supporting Figure

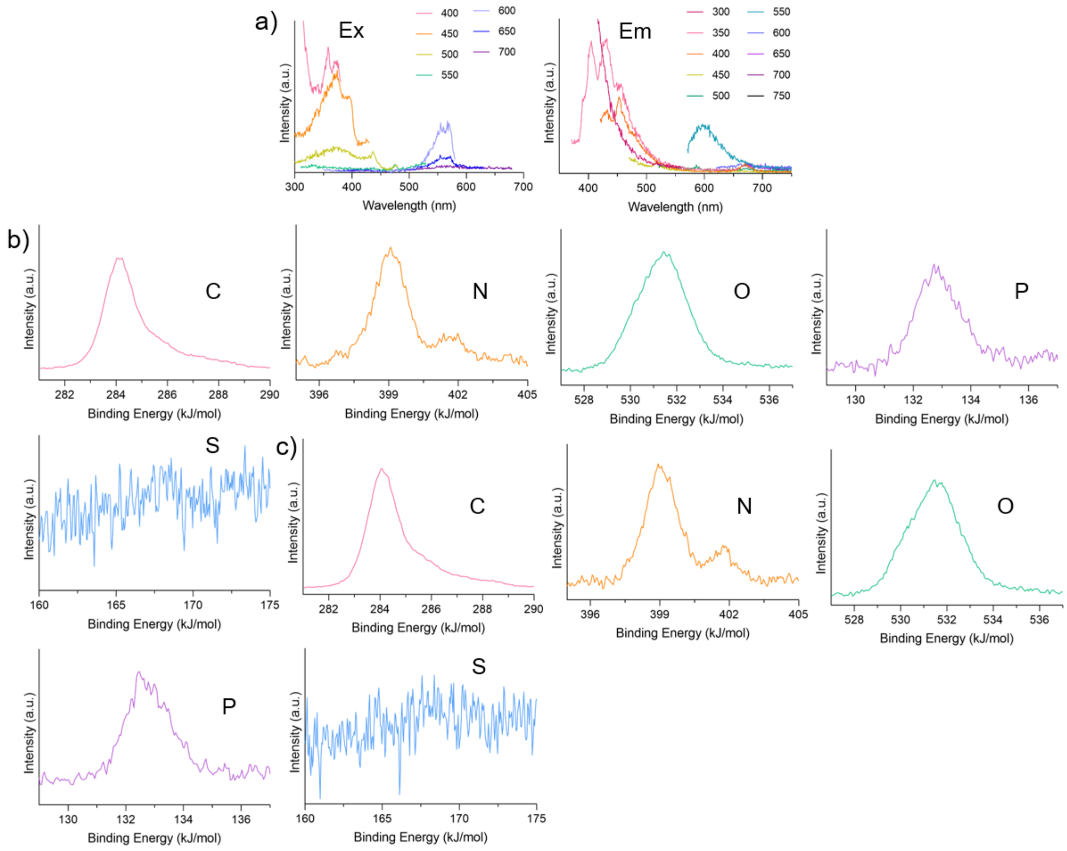

Figure S1. Characterization of Au nanoparticles for intracellular biomineralization. a) Fluorescence excitation and emission spectra (Ex and Em) of Au nanoparticles. The concentration of Auranofin is 100 μM without near-infrared (NIR) irradiation. b-c) High-resolution X-ray photoelectron spectrum (XPS) of Au nanoparticles with b) and without c) NIR irradiation.

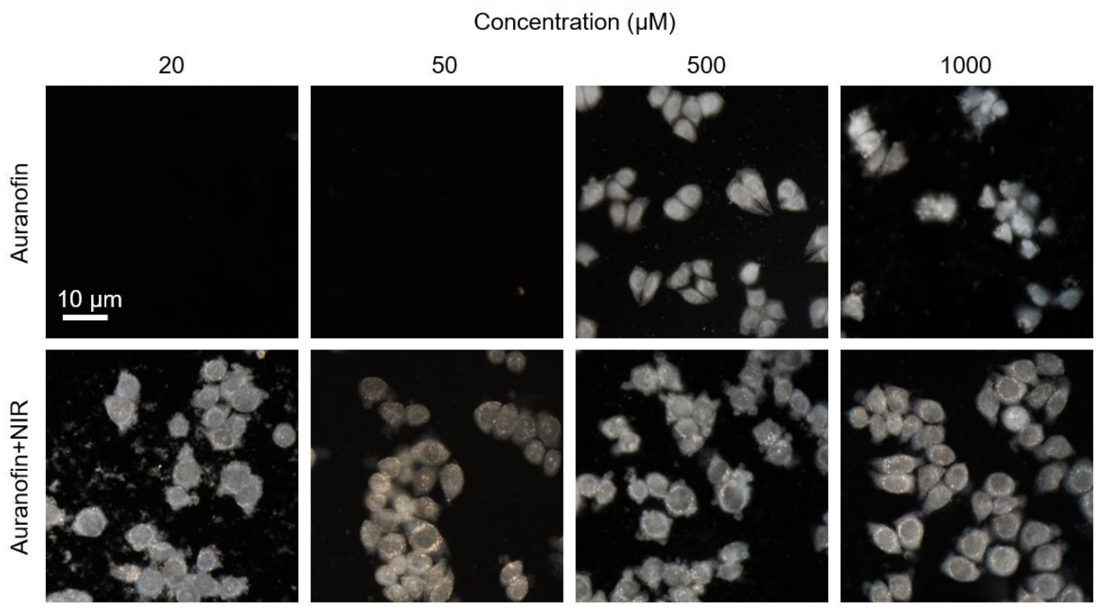

Figure S2. Dark-field microscopic imaging of tumor cells with different treatments.

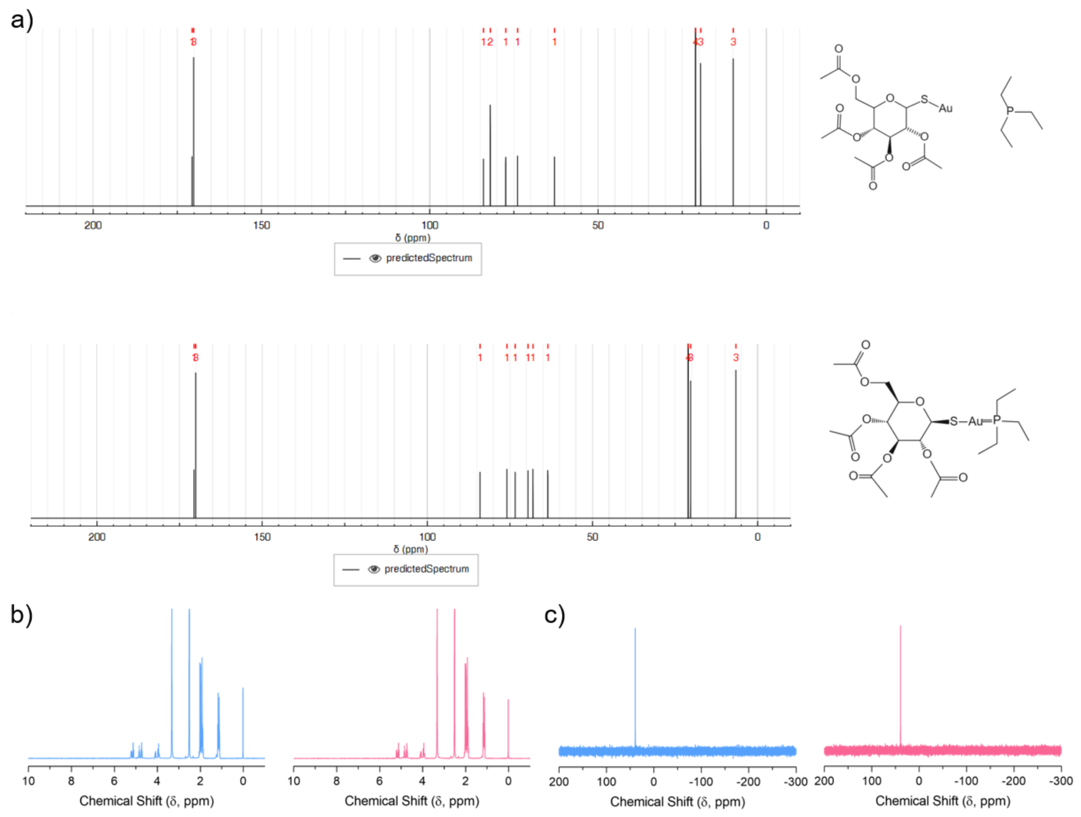

Figure S3. The Nuclear Magnetic Resonance spectrum (NMR) of Auranofin after NIR irradiation . a) Predicted carbon spectrum (^{13}C-NMR). b) Hydrogen spectrum (^{1}H-NMR). c) Phosphorus spectrum (^{31}P-NMR).

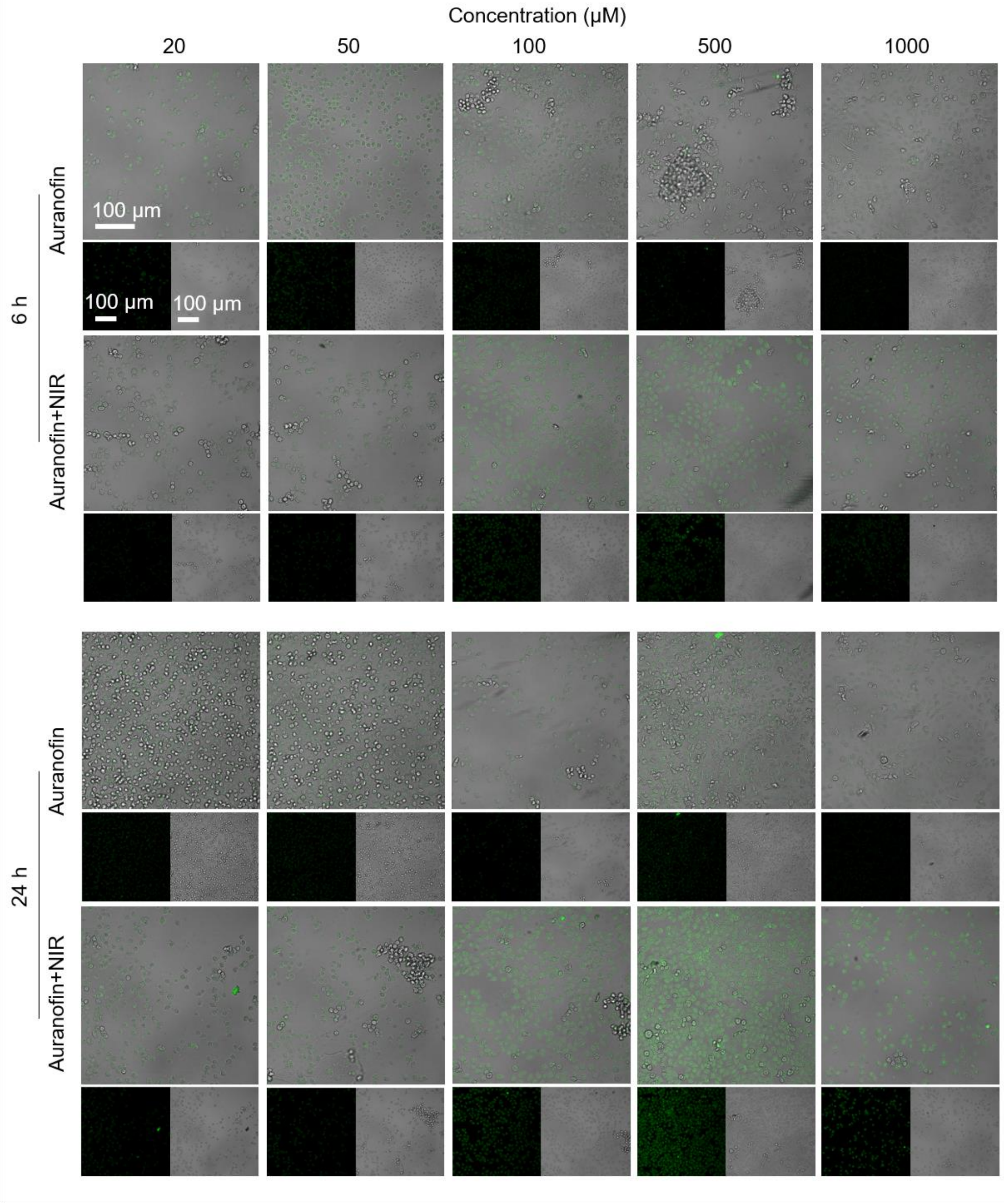

Figure S4. Confocal laser scanning microscopy (CLSM) imaging of tumor cells after treatment with Auranofin of different concentrations at different time points.
